# Supplementary material for: Vinorelbine causes a neuropathic pain-like state in mice via STING and MNK1 signaling associated with type I interferon induction
Source: iScience. 2024 Jan 8;27(2):108808. doi: 10.1016/j.isci.2024.108808 (PMC10831286; doi:10.1016/j.isci.2024.108808)
Supplement: Document S1. Figures S1–S3 [file mmc1.pdf]

iScience, Volume *27*

Supplemental information

Vinorelbine causes a neuropathic pain-like state in mice via STING and MNK1 signaling associated with type I interferon induction

Úrzula Franco-Enzástiga, Keerthana Natarajan, Eric T. David, Krish Patel, Abhira Ravirala, and Theodore J. Price

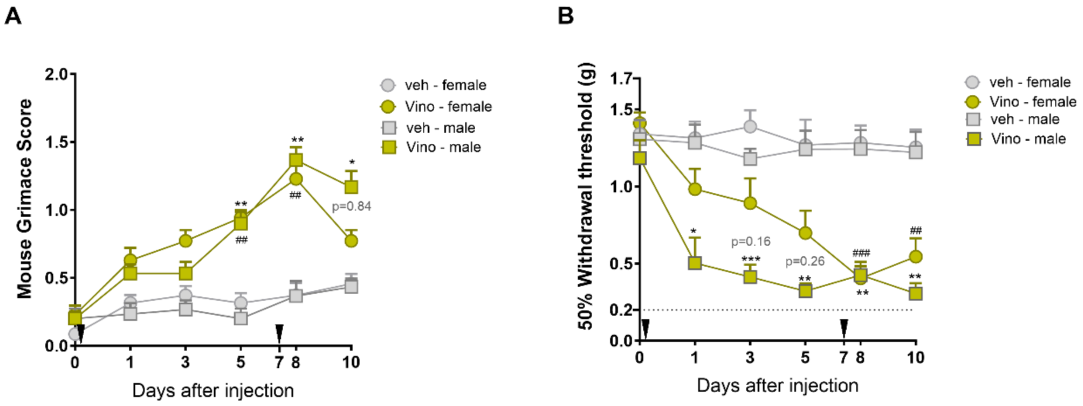

Figure S1. Vinorelbine induces mechanical hypersensitivity and spontaneous pain in both male and female WT ICR mice and sex differences are not observed, Related to Figure 1. **A)** Time course of grimacing score in male and female WT ICR mice at 1, 3, 5, 8, and 10 days after the first administration of vinorelbine (10 mg/kg, i.v.) or vehicle (3% DMSO i.v.). Arrow heads show the time of administration (days 0 and 7). **B**) Time course of mechanical sensitivity in male and female WT ICR mice at 1, 3, 5, 8, and 10 days after the first administration of vinorelbine (10 mg/kg i.v.) or vehicle (3% DMSO i.v.) in paw withdrawal threshold. Data are presented as the mean ± SEM. *p<0.05, **p<0.01, ***p<0.001 vs veh in male animals, and $^{\#\#}p<0.01$, $^{\#\#\#}p<0.001$ vs veh in female animals (n = 7 male and 6 female mice per group). Comparisons between male and female vinorelbine groups are presented by described p values as determined by two-way ANOVA followed by Bonferroni's test. Vino: vinorelbine, veh: vehicle.

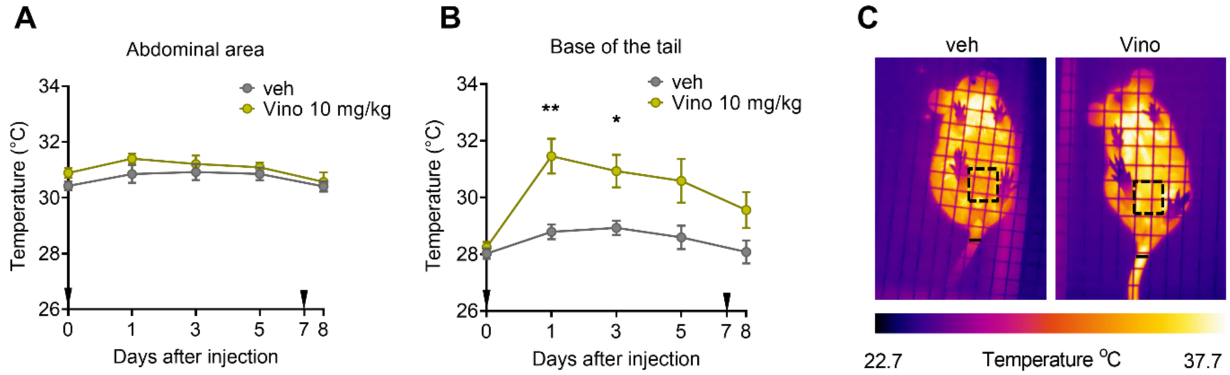

Figure S2. Vinorelbine increases temperature at the site of administration, Related to Figure 1. Changes in temperature either on the abdominal area (**A**) or the site of administration (base of tail, **B**) were assessed before experimental treatment and at 1, 3, 5 and 8 days after the first administration of vinorelbine. Representative colorized infrared thermogram images containing the abdomen wall and tail of mice on day 1 are shown in panel **C**. The color bar represents the temperature in °C. The dotted square represents the abdominal evaluated area, and the straight line represents the selected area considered at the base of the tail. Data are presented as the mean ± SEM. *p<0.05, **p<0.01 (n = 11 per group with 5 male and 6 female mice) as determined by two-way ANOVA followed by Bonferroni's test in **A, B.** Vino: vinorelbine, veh: vehicle.

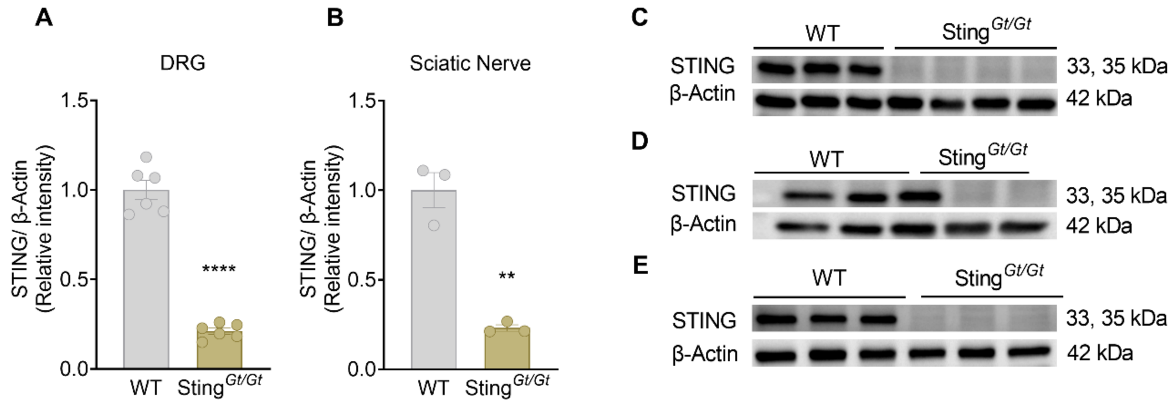

Figure S3. Sting$^{Gt/Gt}$ mice have a robust reduction in STING, Related to Figure 3. Sting$^{Gt/Gt}$ mice have a significant decrease in STING compared to WT mice in DRGs (**A**) as well as sciatic nerve (**B**). Representative western blot images showing STING mean intensity levels in DRGs in Sting$^{Gt/Gt}$ compared to WT male (**C**) and female (**D**) mice. Representative western blot images showing STING mean intensity levels in sciatic nerve in Sting$^{Gt/Gt}$ compared to WT mice (**E**). Data are presented as mean ± SEM. **p<0.01, ****p<0.0001 (n=6 per group in **A**, n=3 per group in **B**), as determined by unpaired t test.
